# Medieval Climate Variability in the eastern Amazon-Cerrado regions and its archeological implications

**DOI:** 10.1038/s41598-019-56852-7

**Published:** 2019-12-30

**Authors:** Vitor Azevedo, Nicolás M. Stríkis, Rudney A. Santos, Jonas Gregorio de Souza, Angela Ampuero, Francisco W. Cruz, Paulo de Oliveira, José Iriarte, Cintia F. Stumpf, Mathias Vuille, Vinícius R. Mendes, Hai Cheng, R. Lawrence Edwards

**Affiliations:** 1 Geochemistry Department, Fluminense Federal University, 24020-141 Niterói, Brazil; 2 Geosciences Institute, University of São Paulo, 05508-0 80 São Paulo, Brazil; 3 Department of Archeology, University of Exeter, Exeter, UK; 4 Geociences Institute, University of Brasília, 70910-900 Brasília, Brazil; 5 Department of Atmospheric and Environmental Sciences, University at Albany, Albany, NY USA; 6 Marine Science Department, Federal University of São Paulo, 11050-020 Santos, Brazil; 7 Institute of Global Environmental Change, Xi’an Jiaotong University, Xi’an, China; 8 Department of Earth and Environmental Sciences, University of Minnesota, Minneapolis, MN USA

**Keywords:** Palaeoclimate, Palaeoclimate, Palaeoecology, Palaeoecology

## Abstract

The South American Monsoon System is responsible for the majority of precipitation in the continent, especially over the Amazon and the tropical savannah, known as ‘Cerrado’. Compared to the extensively studied subtropical and temperate regions the effect of the Medieval Climate Anomaly (MCA) on the precipitation over the tropics is still poorly understood. Here, we present a multiproxy paleoprecipitation reconstruction showing a consistent change in the hydrologic regime during the MCA in the eastern Amazon and ‘Cerrado’, characterized by a substantial transition from humid to drier conditions during the Early (925-1150 C.E.) to Late-MCA (1150-1350 C.E.). We compare the timing of major changes in the monsoon precipitation with the expansion and abandonment of settlements reported in the archeological record. Our results show that important cultural successions in the pre-Columbian Central Amazon, the transition from Paredão to Guarita phase, are in agreement with major changes in the hydrologic regime. Phases of expansion and, subsequent abandonment, of large settlements from Paredão during the Early to Late-MCA are coherent with a reduction in water supply. In this context we argue that the sustained drier conditions during the latter period may have triggered territorial disputes with Guarita leading to the Paredão demise.

## Introduction

In tropical areas, speleothems, secondary cave mineral deposits, are widely employed to reconstruct past variations in the hydrologic cycle^1–6^. Such records allow application of highly accurate and precise dating techniques coupled with geochemical analyses capable of reconstructing past climate variability through development of highly resolved stable isotopic chronologies. Over the Amazon and Central Brazil regions, speleothem records have been used as recorders of past variability of the South American Summer Monsoon (SASM). Some regions, such as the Amazon and Cerrado regions, however, still lack the proper spatial coverage of these high-resolution records. Thus, there is still a need to develop additional paleoclimate records that could provide further insight into how the climate of these regions varied in the past^7–9^. Indeed, δ^18^O variations seen in multiple speleothem records, when combined, can provide a powerful tool to reconstruct the regional-scale climate response to internal and external forcings that affected the South American Continent during the last millenium^10–12^.

One of the main climate events recognized during the last millennium is the Medieval Climate Anomaly (MCA), also known as Medieval Warm Period (MWP). Initially, it was defined as a period of globally increasing temperatures^13^, but over the past decades, several studies, based on temperature-sensitive proxies have reconstructed global and hemispheric temperatures^14^, showing a more complex scenario with rising and decreasing temperatures in different parts of the globe. In South America, this event is most prominently recognized as a dry period over the tropical Andes region, as seen in isotopic records; conversely, in other regions and proxies, its potential impacts are not that well understood^15,16^.

In the last decade, several review studies have been published regarding the South American Summer Monsoon (SAMS) and its climate variability in the last millennium, using high-resolution paleoclimatic records, i.e. speleothems, lake sediments and ice-cores^12,16,17^. Most of these records indicate an increase in precipitation for the Amazon region during the late Holocene^18–26^. However, some of the paleoecological reconstructions are limited in their temporal resolution or dating accuracy and are therefore unable to resolve multidecadal variations over the last two thousand years^27^. Establishing a precise chronology of the timing of multidecadal variations that contributed to periods of wetter and drier climate therefore remains a challenge.

Here, we present two new high-resolution records located in the Eastern regions of the tropical Savannah known as ‘Cerrado’ and the Amazon Forest, respectively, a 23-cm-long stalagmite record from Mata Virgem cave, named MV3 (11°37′27.07″S, 47°29′19.04″W); and the topmost part of core XC01-2, a lacustrine record from Arapujá Lake (3°48′57.56″S, 52°40′27.42″W).

Recently, several studies have addressed the effects of decadal to multi-centennial climate fluctuations in triggering episodes of rise and demise of human civilizations worldwide^28–38^. In the SASM region, the lack of high-resolution paleoclimate and paleoenvironmental records covering the last millennium has hitherto severely limited such studies. Furthermore, far too little is known about how such climate disruptions might have affected past civilizations in the Amazon basin during the pre-Columbian times (before 1492 C.E.). In this context, here we explore the potential of these new proxy records to assess potential links between rise and decline of human settlements and past climate variability in the Amazon basin.

## Pre-Columbian Amazon Archeology

Over the past decades, significant progress has been made in reconstructing the distribution of settlements and population size in the pre-Columbian Amazon^39,40^. Based on recent archeological evidence, Amazonian cultures have been shown to rely on different resources, ranging from intensive agriculture on raised fields, through polyculture in anthropogenic soils, to exploitation of aquatic resources^18,41–44^. During the last millennium, archaeological data reveal widespread migration and conflict across the Amazon, as identified by cultural disruption and fortified settlements^39,45,46^.

**Figure 1:**
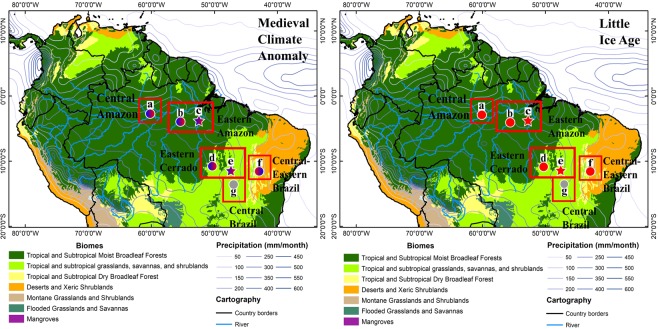
Shaded terrestrial ecoregions^65^ and DJF contoured climatological precipitation in mm/month for the period from 1998 to 2017, with data from TRMM 3b43^66^. The difference between the two scenarios, MCA and LIA, are shown by different color schemes representing the following climate conditions: red - dry; blue-to-red – transition from humid to dry; red-to-blue – transition from dry to humid; grey – neutral conditions. Records are: (**a**) Paredão archeological sites^44^; (**b**) Paraíso Cave^9^; (**c**) Arapujá Lake (this study); (**d**) Bananal Island^56^; (**e**) Mata Virgem Cave (this study); (**f**) DV2 record^53^; (**g**) SBE3/SMT5 records^16^.

One of the better-studied regions where paleoclimatological and archeological data have the potential to enlighten ancient patterns of migration and conflict during periods of climate change is the Central Amazon (Fig. 1a). In this region, two main Amazonian pre-Columbian cultures have been identified, with remarkable differences in terms of their diets and patterns of social-organization: (1) the Paredão phase (645-1250 C.E.), a society which based their diet primarily on fish resources and secondarily on agriculture, and lived in extensive settlements on the margins of rivers in the Amazon Central Region^44,47^, and (2) the Guarita phase (900-1600 C.E.); a society which lived in scattered settlements with smaller extension and a limited permanence of their settlements^48^. While there is still uncertainty regarding the Guarita diet, it is plausible that they had different diets based on the location of the settlements. The Paredão settlements on the other hand represent a more sedentary type of settlement occupation^49^.

Paredão settlements were ring-shaped, with mound architecture, deep accumulation of Amazonian Dark Earth (ADE), and characterized by Incised-Rim ceramic tradition, which is very distinctive from other previous Amazonian occupations^50,51^. They had the largest and most enduring settlements among the archaeological cultures of the Central Amazon, located along the banks of the Amazon and Negro Rivers. While there is no conclusive site identified as the first Paredão settlement, it is believed that they were established in the region around 700 (C.E.)^39,48^. On the other hand, Guarita settlements are associated with polychrome ceramics and were the last pre-Columbian people to survive and occupy the settlements of the Central Amazon before the encounter with European colonizers. Their settlements were scattered from the Amazon Mouth to the Ecuadorian Andes, potentially indicative of long-range migrations, with the first Guarita settlements dated to around the end of the first millennium (C.E.)^39,48^.

For the last decade, there has been considerable debate about why there is no Paredão-phase settlement after the 13^th^ century and why there is hegemony of Guarita settlements at the eve of the European encounter. Evidence of conflicts between those two cultures has been reported^39^ but the reasons that could have triggered this conflict and the eventual disappearance of the Paredão from the archeological record are still debated and speculative. Given the proximity of settlements to rivers in the Central Amazon, and the predominance of aquatic fauna in the diet of lower Amazonian cultures^43,44^, the availability of and dispute over aquatic resources may have been a prime cause of the cultural disruptions and conflict evidenced in the archaeological record of the last millennium.

However, so far no paleoclimatic and paleoecological evidence for changes in hydrologic conditions during these different periods of occupation has been presented for the Eastern Amazon and Cerrado regions. Here we document that anomalously humid conditions prevailed during the Early-MCA around (925-1150 C.E.), followed by a less humid period (1150-1350 C.E.). These changing climatic conditions may have strongly affected the pre-Columbian Amazon population, their settlements and, possibly contributed to migration and their disappearance during the first half of the last millennium.

## Results

**Figure 2:**
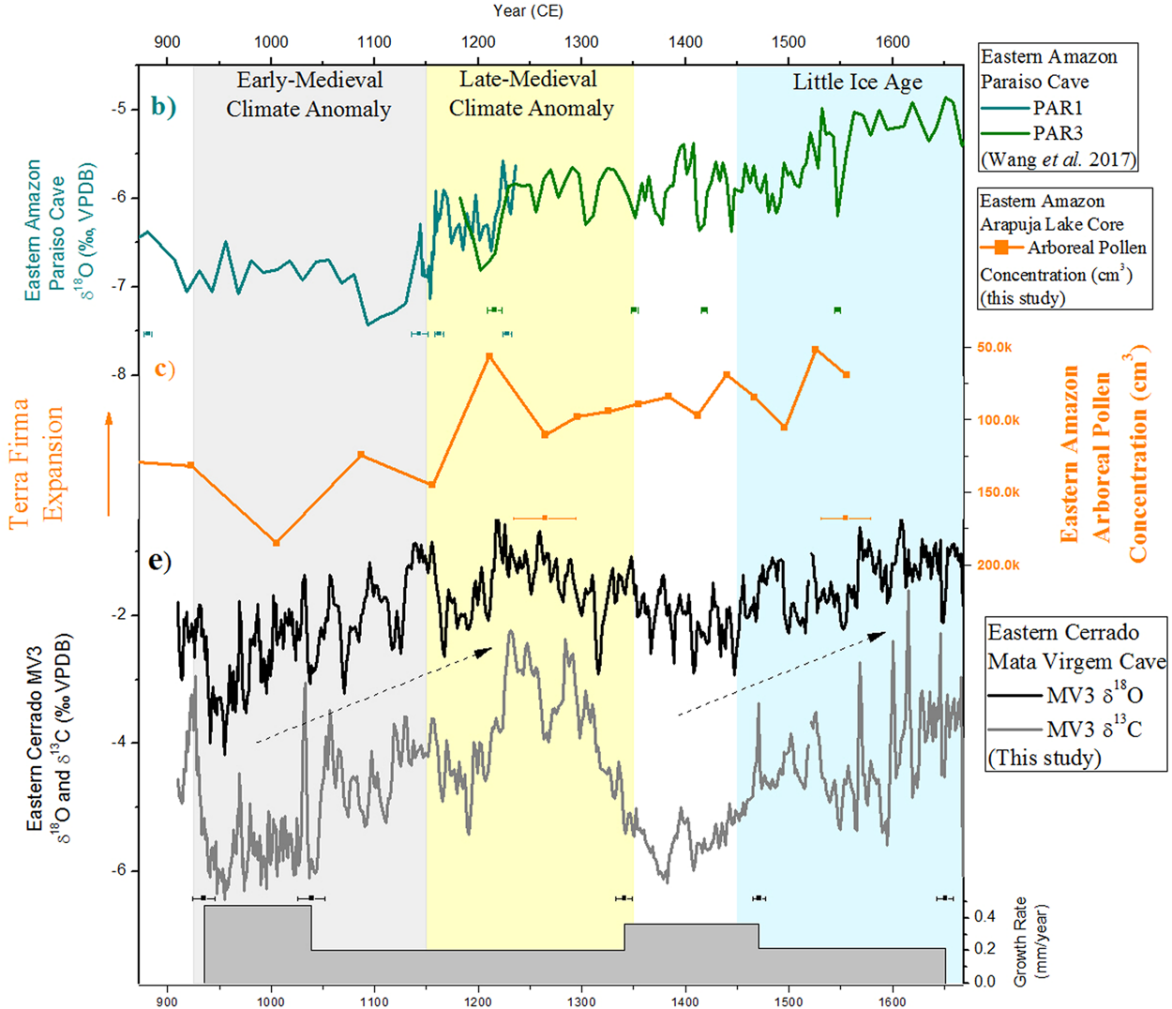
Comparison between Eastern Amazon and Eastern Cerrado records. (**b**) Paraíso Cave – PAR1 and PAR3 records – Eastern Amazon^9^, (**c**) Arapujá Lake Arboreal palynomorphs concentration (cm^3^) from lacustrine core – Eastern Amazon (this study) and (**e**) Mata Virgem cave – MV3 δ^18^O, δ^13^C and growth rate records - (this study) – Eastern Cerrado.

The MV3 stalagmite grew from 910 to 1680 C.E. (Fig. 2e) with growth rates between 0.20-0.47 mm/year (Fig. S1). It was sampled at a resolution of 0.4 mm for δ^18^O and δ^13^C analysis. The δ^18^O record varies between -0.44 to -4.17 per mil (‰) and is characterized by multidecadal and centennial-scale variations throughout this period. The δ^13^C record varies between -1.60 to -6.44 per mil (‰) and is characterized by two persistent negative excursions followed by increasing trends. Here, based on the IPCC definitions of Medieval Climate Anomaly (ca. 950-1250 C.E.) and Little Ice Age (ca. 1450-1850 C.E.)^52^, we sub-divide the record into the following periods: The Early-Medieval Climate Anomaly (E-MCA) from 925-1150 C.E., with the lowest values of δ^18^O and δ^13^C and higher growth rate, sustained for a little more than two centuries; and the Late-Medieval Climate Anomaly (L-MCA) from 1150–1350 (C.E.), with the highest values of δ^18^O and δ^13^C and lower growth rate, also lasting for about two centuries. Following the L-MCA phase, the MV3 record is characterized by minor negative fluctuations, with slightly more negative δ^18^O values and higher growth rate and more depleted δ^13^C values during the decades between 1350–1450 (C.E). Afterwards, the δ^18^O and δ^13^C increases again and growth rate slows, reaching similar positive values during the LIA (1450–1850 C.E.) as the previous L-MCA period. The transition from lower to higher δ^18^O and δ^13^C values during the E-MCA to L-MCA transition, seen in the MV3 stalagmite, is consistent with a major shift in the pollen concentration of arboreal palynomorphs recorded in the Arapujá Lake, dropping from a maximum of 185,026 to a minimum of 56,463 pollens/cm^3^ (Fig. 2c). This period covering the last millennium is marked on the top most part of the Arapujá lake record (Fig. S5). Here we show the last two of four zones from the record: the zone III - covering the period of 560–1211 C.E. (1390–739 B.P.); and the zone IV covering the period of 1211–1440 C.E. (739–510 B.P.). The four zones can be seen in the Supplementary Material (Figs. S3–S5). During pre-LIA period, the zone IV in Arapujá Lake, some taxa appear only in minor concentrations and others even disappear completely, ending the record with a decreasing trend.

## Discussion

**Figure 3:**
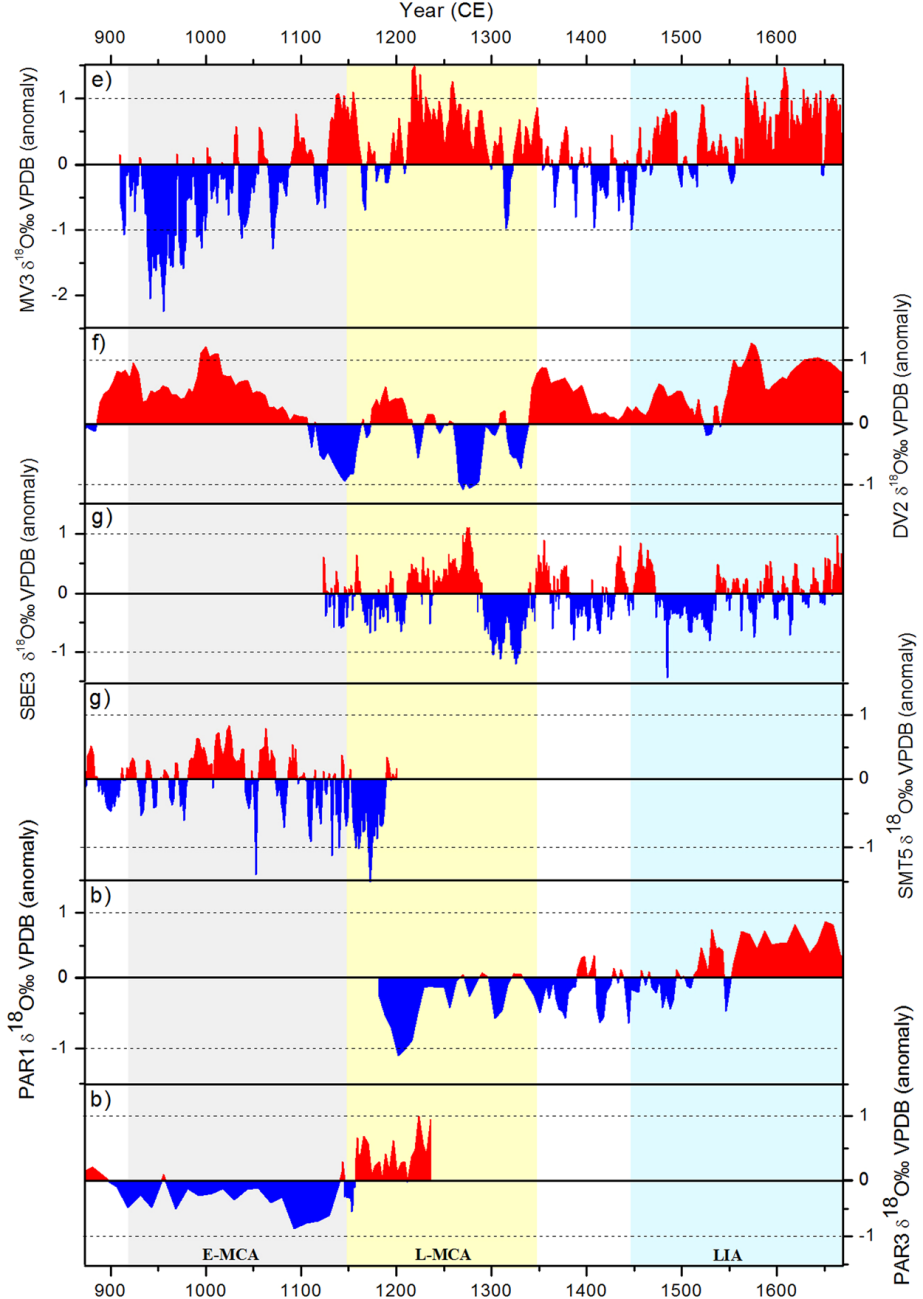
Comparison of δ^18^O anomalies from stalagmite records during the last millennium. Records include from top to bottom: (**e**) MV3 δ^18^O record– Eastern Cerrado (this study); (**f**) Diva de Maura Cave – DV2 δ^18^O record – Central-Eastern Brazil^53^; (**g**) SBE3 – São Bernardo Cave and SMT5 – São Mateus Cave δ^18^O record – Central Brazil^16^; (**b**) PAR1 and PAR3 – Paraiso Cave δ^18^O record – Eastern Amazon^9^. The anomalies were calculated subtracting the average δ^18^O isotope value from each record.

The novel paleoclimate record presented here shows a consistent scenario of a sustained humid period and higher vegetation-soil productivity during the E-MCA (925–1150 C.E.) over the Eastern ‘Cerrado’ region (Fig. 2e), based on lower values of δ^18^O and δ^13^C and higher growth rate from MV3 stalagmite. The wet phase identified in the speleothem record from Mata Virgem cave is contemporaneous with a high initial arboreal palynomorphs concentration extracted from Arapujá Lake in the Eastern Amazon region. The subsequent transition from humid to drier conditions and lower vegetation-soil productivity during the L-MCA (1150–1350 C.E.), indicated by higher values of δ^18^O and δ^13^C and lower growth rate, are accompanied by a reduction in arboreal pollen concentration (Fig. 2c). This two-phase tendency during the MCA was not seen in previous stalagmite records from other parts of Brazil with exception of the Paraíso Cave records^16,53,54^. Furthermore, this transition during the MCA (Fig. 3e) is opposite to those found in Central (Fig. 3g) and Central-Eastern (Fig. 3f) Brazilian stalagmite records. This suggests more humidity transported from the Eastern Amazon over the Eastern Cerrado during the Early-MCA.

Previous stalagmite records from the South American Monsoon System (SAMS) region demonstrated that the amount effect is the predominant factor controlling the δ^18^O variations during the last two millennia^12,16,55^. This means that negative (positive) values represent periods of higher (lower) precipitation for the region. Additionally, in the case of the MV3 stalagmite, more negative δ^13^C values and higher growth rates are coherent with the two periods of more negative δ^18^O values during the Early-MCA and pre-LIA, with the first one being the more negative of the two, suggesting a correlation between low δ^13^C and high growth rate with higher vegetation-soil productivity.

Furthermore, a good correlation exists between interannual rainfall variability and stalagmite δ^18^O over the last two centuries in Sao Bernardo cave stalagmites^55^. This calibrated cave record is located around 380 km south of our studied site. Both regions present similar vegetation cover (Fig. 1) and epikarst depth. Additionally, 2012–2018 seven-days back trajectories from Paraiso, Mata Virgem and Sao Bernardo/Sao Mateus Cave locations showed similar trajectories, especially during the strong monsoon season (Fig. S6).

**Figure 4:**
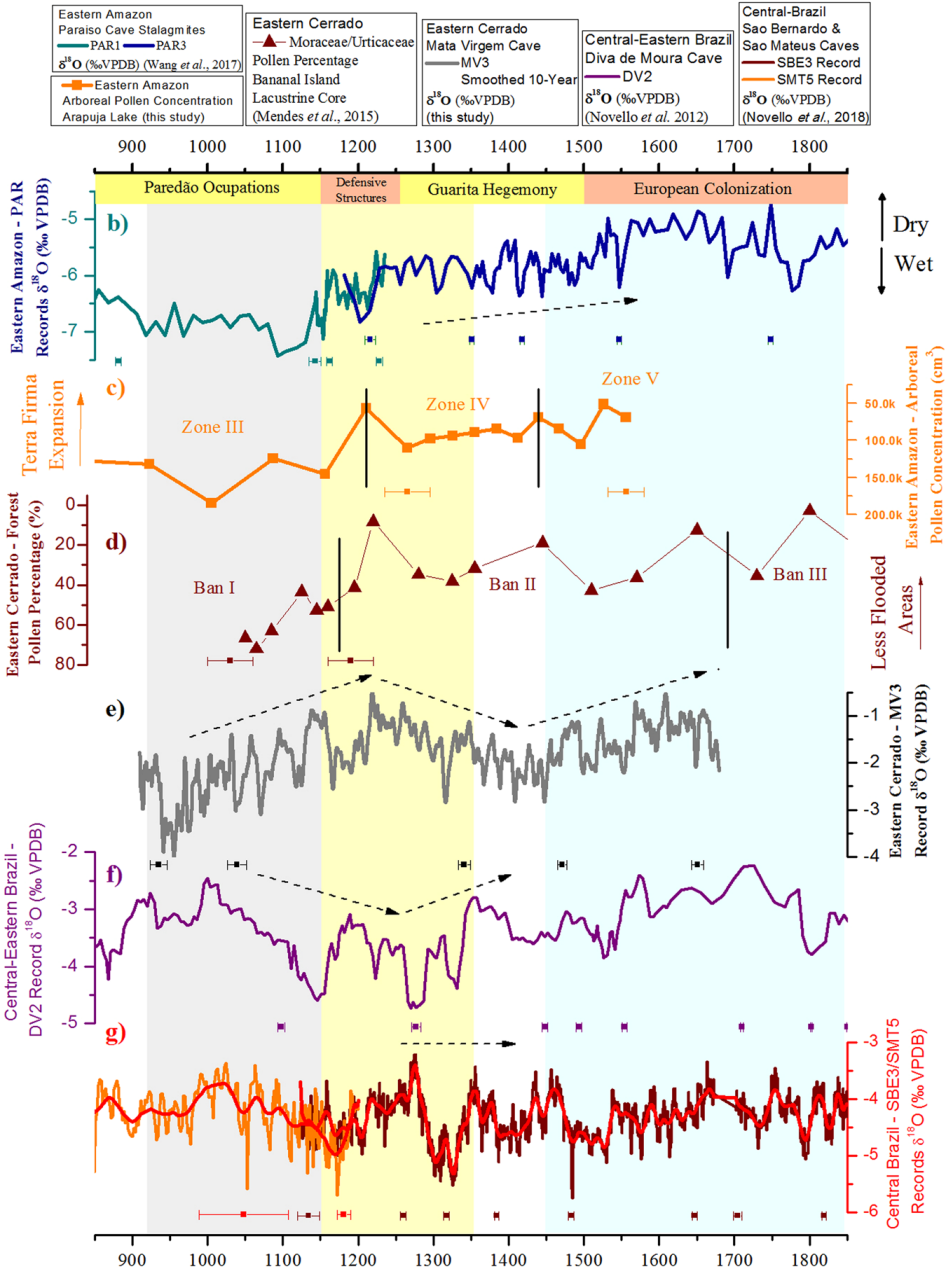
Comparison of paleoclimate and paleoecological records during the last millennium. Paleoecological zones indicated are as determined by the authors of the respective publications. Records include: (**b**) Paraíso Cave – PAR 1 and PAR3 δ^18^O records – Eastern Amazon^9^; (**c**) Arapujá Lake Arboreal pollen concentration (cm^3^) - Eastern Amazon – (this study); (**d**) Bananal Island Forest Pollen Percentage – Eastern Cerrado^56^; **(e**) Mata Virgem Cave – MV3 δ^18^O record – Eastern Cerrado (this study); (**f**) Diva de Moura Cave - DV2 δ^18^O record – Central-Eastern Brazil^53^; (**g**) São Bernardo and São Mateus Caves -SBE3/SMT5δ^18^O records – Central Brazil^16^. Periods of occupation from different cultures shown are based on ^14^C-dated materials in Central Amazon sites^39,44,48,51,58^.

In order to help identify whether the humid period extended to other neighboring areas during the Early-MCA, we compare the (e) MV3 record and a (c) high-resolution last millennia lacustrine pollen recordfrom the Eastern Amazon to: (b) stalagmite records from Paraíso Cave in the Eastern Amazon Region^9^; (f) a stalagmite record from Diva de Moura Cave, in the Central-Eastern Brazil region^53^; (g) stalagmite records from São Bernardo and São Mateus caves, in the Central Brazil region^16^; and (d) a high-resolution pollen record from Bananal Island, in the Eastern Cerrado region (Fig. 4).

The combined speleothem and pollen record from Eastern Amazon-Cerrado regions presented here suggests that the climatic variations related to the MCA were particularly relevant for the climate and the environment of the region during the last millennium. Speleothem records from Paraíso Cave^9^, together with other paleoclimate records, located in the Eastern-Cerrado region^56^ suggest the same coherent pattern for E-MCA (strong humid period) followed by L-MCA (drier period). Minor oscillations in the speleothem isotope record show a decoupling between MV3 and the Arapujá lake record, evidenced by the sustained reduction in the concentration of arboreal pollen after the L-MCA while the MV3 δ^18^O record reaches slightly more negative values during the decades of 1350–1450. Starting at the beginning of the LIA period, around 1500 (C.E.), trends are again similar between the MV3 record and Arapujá Lake. Diminishing concentrations of arboreal palynomorphs can be related to larger exposed areas of grasslands that might have been previously flooded.

During the E-MCA, speleothems records from (f) Central-Eastern Brazil (Diva de Maura cave)^53^ and (g) Central Brazil (São Bernardo and São Mateus Cave)^16^ show opposed tendencies when compared to the (e) MV3 and (c) Arapujá Lake records (Fig. 4), with the former being characterized by negative trends in δ^18^O, reaching relatively low values that are sustained during the L-MCA. During the period pre-LIA, when MV3 seems to be decoupled from Arapujá Lake and Paraíso cave records, the trends between the Eastern Cerrado and Central-Eastern Brazil are similar. Thereafter, our MV3 record, along with (b) Paraiso and (f) Diva de Maura, show an increase in the δ^18^O during the LIA portraying a consistent scenario of dry conditions during the LIA over these regions. According to Novello *et al*., (2018), a clear humid phase is recorded during the LIA period in southward-located stalagmites within the SAMS region, while drier and neutral conditions are found within northward-located stalagmites, i.e. from (f) the Central-Eastern and (g) the Central Brazil regions^16^. Our results suggest an extended period of drier conditions during the LIA within the Eastern Cerrado-Amazon regions (Fig. 1).

Additional evidence for an anomalous humid period in the Eastern Cerrado during the E-MCA stems from the Bananal 8 pollen record^56^. This record is based on a lacustrine core, about 450 km west from Mata Virgem Cave. This record documents an event that overlaps with the E-MCA in MV3, apparent between 1030–1180 C.E. (BAN I; Fig. 4d). The BAN-I event is associated with higher percentages of forest taxa, especially an increased occurrence of *Moraceae/Urticaceae*. After this period, forest pollen (*Moraceae*/*Urticaceae*) decreased until the middle of the LIA period, defining a second zone, called BAN-II. The third zone, or BAN-III, begins once forest pollen reaches a new relative low in the record. The transition from high to low forest pollen percentage goes hand in hand with higher concentrations of herbs and grass pollen due to an expansion of Terra Firma and grasslands, which had been flooded during the previously humid phase.

In summary, based on the two records of this study, along with other paleoclimatic records for the Eastern Amazon and Cerrado regions, it was possible to define three distinct periods for the pre-industrial last millennium:

(1) The Early-Medieval Climate Anomaly (925–1150 C.E.), represents a pronounced humid period, with the lowest δ^18^O and δ^13^C values and highest growth rate recorded in the MV3 stalagmite along with the highest arboreal palynomorphs concentration found in the Eastern Amazon Arapujá Lake core; (2) During Late-Medieval Climate Anomaly (1150–1350 C.E.), arboreal palynomorphs concentrations in the Eastern Amazon Arapujá Lake core indicate sustained drier conditions, consistent with the higher δ^18^O and δ^13^C values and the lowest growth rate in the Cerrado MV3 stalagmite; (3) Before and during the Little Ice Age (starting at 1350 C.E.), MV3 δ^18^O and δ^13^C values start to increase and growth rate begins to decrease after a period of relative lower isotope values and high growth rate, reaching similar values during the LIA as those seen during the dry L-MCA, dropping off only at the very end of the record (1669 C.E.). Similarly, the Eastern Amazon Arapujá Lake sustains the decreasing trend of arboreal palynomorphs concentration until the end of the record (1556 C.E.), at the beginning of the LIA.

## Archeological Implications

Paredão diet was shown to rely, to a large extent, on aquatic resources. At the Hatahara archeological site (Fig. 1a), in the Central Amazon^44^, the presence of seasonal species indicates a population specialized in the capturing of specific fish, during events of annual flooding, with around 90% of the species captured in aquatic or semi-aquatic environments. This specialization may have favored the expansion of settlements related to the Paredão phase during the E-MCA, and the absence of flooding events during the L-MCA could have significantly affected the old strategies and procurement of aquatic resources in these settlements.

We propose that, around the year 1000 (C.E.), the pronounced increase in population and occupation of the Paredão phase occurred due to the favorable strong humid period during the E-MCA (Fig. 4). Furthermore, new paleobotanical evidence suggests that the role of agriculture has been over-estimated for some communities in these riverine settlements of the Amazon^57^. Stronger humid conditions during the E-MCA probably boosted the fishery-based Paredão settlements. This situation likely changed during the L-MCA, when higher Paredão population and increasing demand for aquatic resources were no longer sustainable. At the same time, this period is characterized by Guarita populations’ dispersion to new areas of non-flooded sites, establishing settlements in regions which flood-adapted populations previously inhabited. The conflict between these cultures, during the E-MCA, eventually led to a domination of Guarita over Paredão, evidenced by the cultural shift of ceramics in the archeological record from the 13^th^century (C.E.). Additionally, the building of defensive structures between the 10^th^ and 13^th^ centuries^39^, around the same time when the Guarita population spread out over the Amazon region^48,50,58^, reinforces the situation of conflict, direct or indirect, during this period of transition.

The agreement between the paleoclimatic records and the archeological evidence suggests a coherent regional climate signal over the Eastern, Central and bordering regions of the Amazon during at least the last millennium. The changing climate during the MCA, likely affected the strategies and occupation of different pre-Columbian people, potentially impacting routes of migration and settlement coherence.

Additionally, several studies in the last decades suggest that minor hydrological fluctuations could have triggered collapses to even complex and stable civilizations, such as the Mayan, Chinese, Middle East and Indian civilizations^28–30,34–38,59^. Thus, it is plausible that minor hydrological fluctuations may have affected less complex societies as those found in the Amazon during the MCA.

## Conclusion

In this study we present new evidence for a strong humid period in the Eastern Amazon-Cerrado region during the Medieval Climate Anomaly, which we term the Early-Medieval Climate Anomaly (925–1150 C.E.). This humid period is followed by drier conditions during the Late-Medieval Climate Anomaly (1150–1350 C.E.).

The Early to Late-MCA transition from a pronounced humid phase to drier conditions seen in the MV cave, Paraíso cave and Arapujá lake records, seems antiphased with other paleoclimatic records from the Central-Eastern and Central Brazil. However, this apparently antiphased tendency changes between the Late-MCA and the onset of LIA, when all archives tend to record less humid conditions than during the Early-MCA.

The humid E-MCA in the Eastern Amazon and Cerrado likely had implications for populations’ development of pre-Columbian people living in the region and its surroundings. During the Early-MCA, the humid conditions might have contributed to the expansion and establishment of one of the largest fishery-based settlements by groups from the Paredão phase. During the L-MCA, the transition to a drier period probably led to instability among these large groups, potentially triggering internal and external conflicts between the Paredão and another Amazon group, the Guarita phase. The location of the riverine settlements was extremely important for Paredão groups, as their diets were mostly based on species captured in aquatic and semi-aquatic environments, lower levels of river and lacustrine environments. Hence the forced abandonment of the settlements consequently led to dispute over space with other groups, such as the Guarita culture, in the Central Amazon. This conflict, evidenced by construction of defensive structures, might have forced the migration of the Paredão out of this region, and led to its demise and disappearance from the archeological record.

In summary, a drier phase, starting during the L-MCA and lasting beyond that period, may have acted as a catalyst, resulting in the collapse of the Paredão settlements, which are no longer found after the 13^th^ century. Guarita people eventually reoccupied some settlements abandoned by the Paredão until the European Colonization. While there were several other contributing factors for this conflict, climate change might have acted as one important cause for the instability of these groups during the last millennium. This study highlights the potential of high-resolution and well-dated paleorecords for exploring past variations of the South American Summer Monsoon, and how these archives can contribute to archeological studies in this region, by providing information about the effects of climate variability on past civilizations.

## Methods

### Dating

U-Th measurements on MV3 were realized using a multi collector inductively coupled plasma mass spectrometry (Thermo-Finnigan NEPTUNE) at the Institute of Global Environmental Change, Xi’an Jiaotong University, China. A total of 7 samples were dated according to Cheng *et al*. (Table S1)^60^. Analytical precision for the age-model varied from ±6 to ±19 years. The stalagmite growth was continuous, varying from 0.1 to 0.47 mm/year throughout the period analyzed from 873 to 1669 (C.E.). A linear-age model was used to construct the age-model based on 5 samples (Fig. S1).

A total of four ^14^C date measurements were obtained from the Arapujá Lake core and analyzed at 20 cm depth at the AMS Radiocarbon Laboratory, Federal Fluminense University, Brazil and at 40, 60 and 80 cm depths at the Beta Analytics Laboratory, Florida, United States. The ages were calibrated following the SH cal 13 curve^61^ (Table S2). The age-model was produced using the R-Bacon package^62^ (Fig. S2).

### δ^18^O and δ^13^C measurements

Oxygen and Carbon stable isotopes were measured in the Isotope Ratio Mass Spectrometer at the Stable Isotopes Laboratory, University of Sao Paulo, Brazil. A total of 538 samples were analyzed to construct the δ^18^O and the δ^13^C MV3 record at a near-annual resolution varying between 0.8–2 samples/year. The δ-notation is based on the VPDB (Vienna Pee Dee Belemnite) standard for carbonate samples. For the oxygen, the formula is δ^18^O = [((O18/O^16^) sample/(O^18^/O^16^) VPDB) – 1] × 1000 (per mil) and δ^13^C = [((C^13^/C^12^) sample/(C^13^/C^12^) VPDB) – 1] 2 1000 (per mil).

### Lacustrine records

The Arapujá Lake pollen grain analyses were realized according to the protocol of Colinvaux *et al*.^63^. All analyses were performed at the Laboratory of Micropaleontology at the University of Sao Paulo, Brazil. For each sample, around 200 terrestrial pollen grains were counted. Details of the procedure are discussed in the Supplementary Material (Methods S1). Additionally, the data from other paleoclimatic records used in this study were manually extracted using the online version of WebPlotDigitizer^64^.

## Supplementary information

Supplementary Material.

## Data Availability

The dataset generated as part of this study will be archived in the NOAA Paleoclimatology Database.
