# Supplementary material for: Medieval Climate Variability in the eastern Amazon-Cerrado regions and its archeological implications
Source: Sci Rep. 2019 Dec 30;9:20306. doi: 10.1038/s41598-019-56852-7 (PMC6937329; doi:10.1038/s41598-019-56852-7)
Supplement: Supplementary file 1 — Supplementary Material. [file 41598_2019_56852_MOESM1_ESM.docx]

Supplementary Information for

**Medieval Climate Variability in the eastern Amazon-Cerrado regions and its archeological implications**

**Vitor Azevedo^1,*^, Nicolás M. Stríkis^1^, Rudney A. Santos^2^, Jonas Gregorio de Souza^3^, Angela Ampuero^1^, Francisco W. Cruz^2^, Paulo de Oliveira^2^, José Iriarte^3^, Cintia F. Stumpf^4^, Mathias Vuille^5^,Vinícius R. Mendes^6^, Hai Cheng^7,8^, R. Lawrence Edwards^8^.**

^1^Geochemistry Department, Fluminense Federal University, 24020-141 Niterói, Brazil; ^2^Geosciences Institute, University of São Paulo, 05508-080 São Paulo, Brazil; ^3^Department of Humanities, Universitat Pompeu Fabra, 08002 Barcelona, Spain; ^4^Geociences Institute, University of Brasília, 70910-900 Brasília, Brazil;^5^Department of Atmospheric and Environmental Sciences, University at Albany, State University of New York;^6^Marine Science Department, Federal University of São Paulo, 11050-020 Santos, Brazil; ^7^Institute of Global Environmental Change, Xi’an Jiaotong University, 710049 Shaanxi, China; ^8^Department of Earth Sciences, University of Minnesota, Minneapolis, MN 55455. Correspondence and requests for materials should be addressed to V.A.A. (*email: [vitorazevedoalves@gmail.com](mailto:vitorazevedoalves@gmail.com))

**This PDF file includes:**

Methods S1 and S2

Figs. S1 to S6

Tables S1 to S2

Methods

Pollen Analysis (S1)

The sediment samples for the palynological analysis were chemically treated in the Micropaleontology Laboratory of the Department of Sedimentary and Environmental Geology (DGSA) of the Institute of Geosciences of the University of São Paulo, according to the technique described in Colinvaux et al. (1999)^1^ that uses the following steps:

• 1 cm^3^ sub-samples were obtained and 1 tablet of the exotic *Lycopodium clavatum* was added with a known concentration (20,848 grains). The sub-samples were stored in centrifuge tubes and all subsequent chemical steps were centrifuged at 3000 RPM for 5 minutes, and between each chemical reagent the sub-samples were washed with water to stop the chemical reaction.

• 5 ml of 10% hydrochloric acid (HCL) for 10 minutes;

• 5 ml hydrofluoric acid (HF) 100 ° C for 2 hours;

• 5 ml 10% potassium hydroxide (KOH) for 10 minutes;

• 5 ml glacial acetic acid (CH_3_COOH) to remove water from the sample, as the next step can be explosive in contact with water;

• 5 ml of the solution composed of 9 parts of acetic anhydride (CH_3_CO) _2_ and 1 part of sulfuric acid (H_2_SO_4_) for 10 minutes;

• 5 ethyl alcohol (C_2_H_5_OH).

After processing the final residue is stored in glycerin and slides were prepared and sealed with paraffin. The identification, description, and counting of the pollen grains and spores were performed under an optical microscope with a digitized image capture system. The analyzes are performed under magnification of 400x and 1000x (immersion in oil).

For the identification of the pollinic types, the technique of comparison of the palynomorphs found in the sediments with pollen grains and spores kept in a modern reference collection and by queries to the specialized literature^1–3^ among others. During the palynological work, 200 grains of terrestrial pollen per sample were counted (arboreal and herbs), aquatic herbs, spores and algae taxa do not enter this count. The data were treated pre-statistically through the specific programs TILIA, TILIAGRAPH^4^ and CONISS^5^. The latter allows the delimitation of paleoecological zones in the final diagrams, which characterize local environmental changes.

**Back-trajectories (S2)**

The Air parcel back-trajectories from Mata Virgem and Paraiso cave’s locations were modeled with the HYbrid Single-Parcel Lagrangian Integrated Trajectory 4 (HySPLIT 4) model^6,7^. Wind fields from 1500 meters above ground level (m.a.g.l.) come from Era Interim on native spatial resolution of 0.75°. The times for trajectories were from May 2012 to May 2018 at 12 UTM. The period of seven days were used due to the mean residence time of water vapor in the atmosphere^8^.


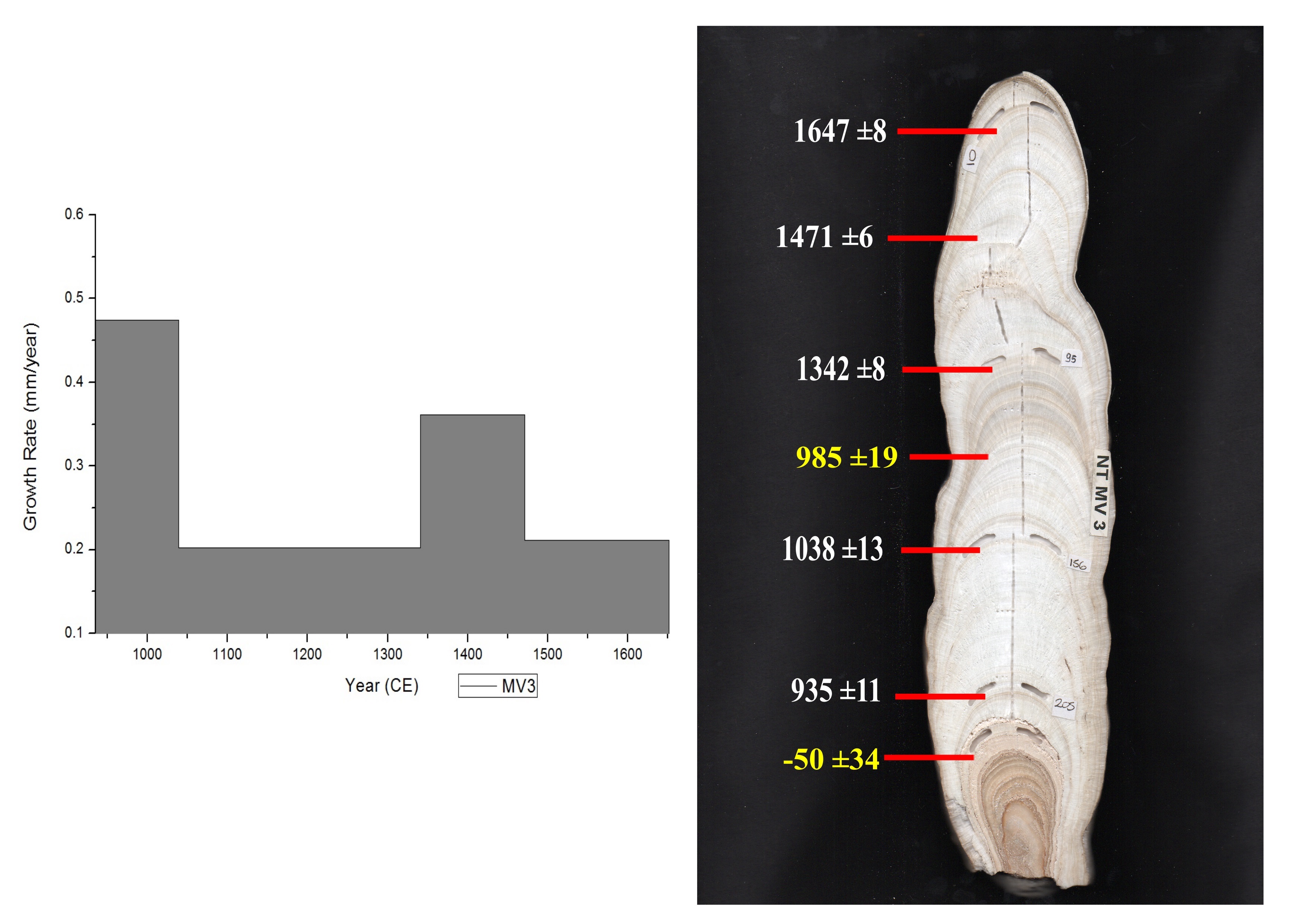


**Figure S1.** MV3 stalagmite growth rate varying between 0.20 and 0.47 mm/year (left) based on a linear age-model. Scanned sample with U-Th age results and 2σ errors. Yellow age results were not used for the model duet to age inversion and hiatus at the bottom of the sample (right).


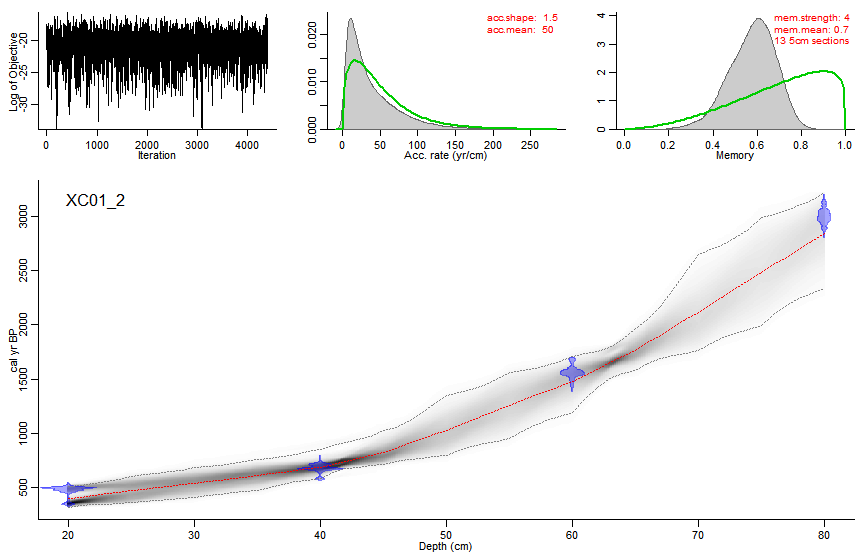


**Figure S2.** Age model of Arapujá Lake created in package Rbacon^9^. The ^14^C results for Arapujá Lake core are between 2,844 and 394 cal years. (B.P.), and the age model generated from these results.


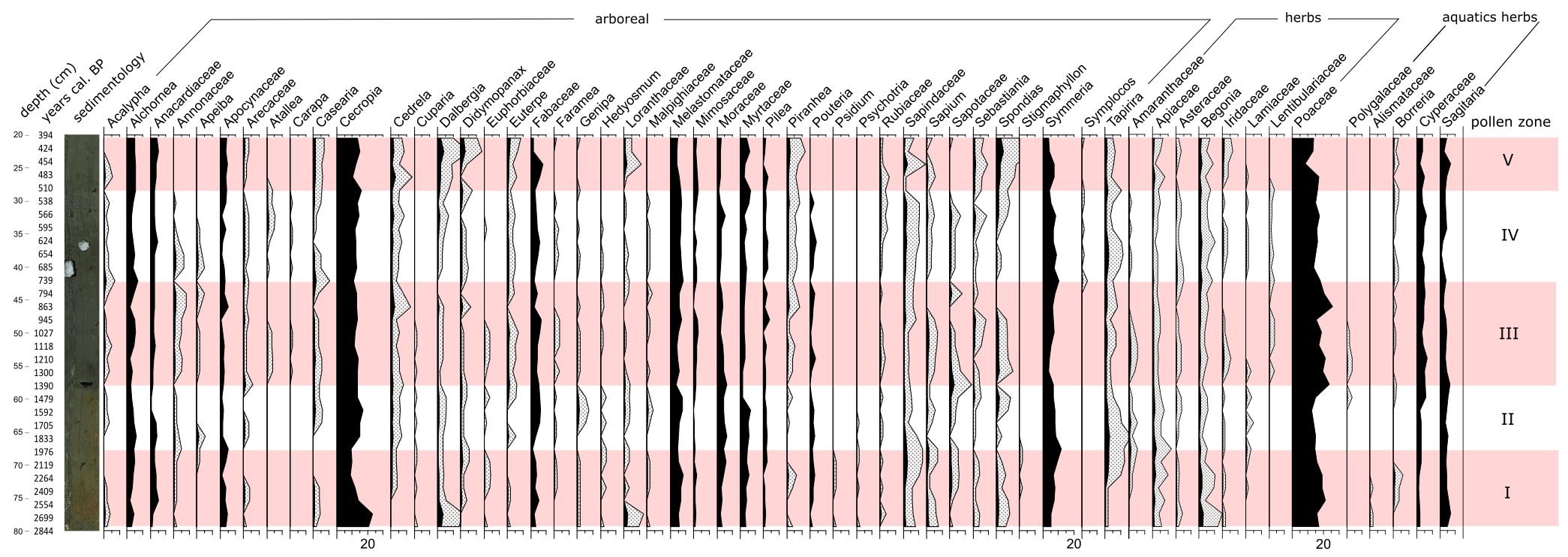


Figure S3. Diagram percentage between arboreal, herbs and aquatic herbs taxa of Arapujá Lake. In pink and white the five pollen zones.


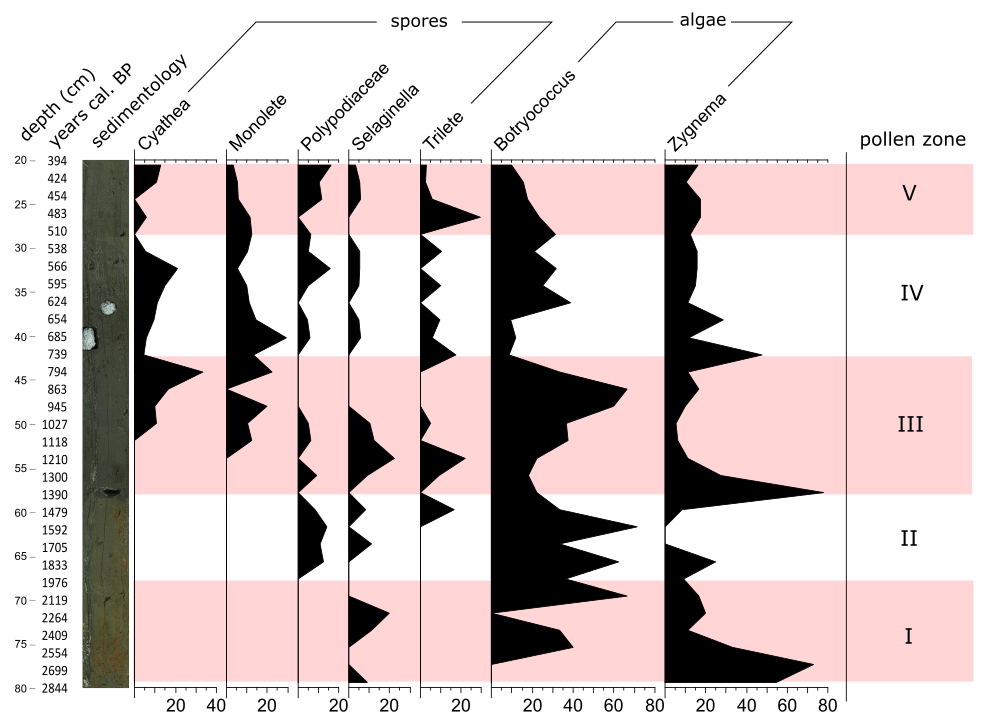


Figure S4. Diagram of percentage between spores and algae taxa of Arapujá Lake.


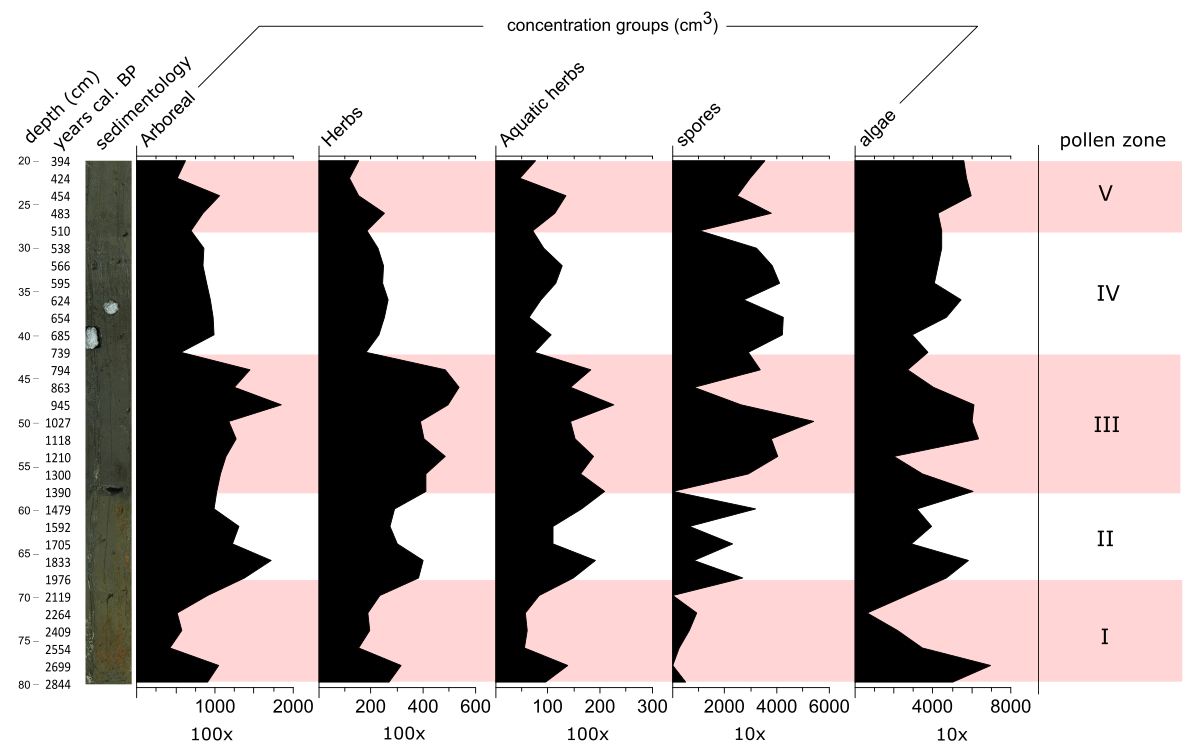


Figure S5. Diagram of concentration (cm^3^) of the pollinic groups arboreal, herbs, spores and algae of Arapujá Lake.


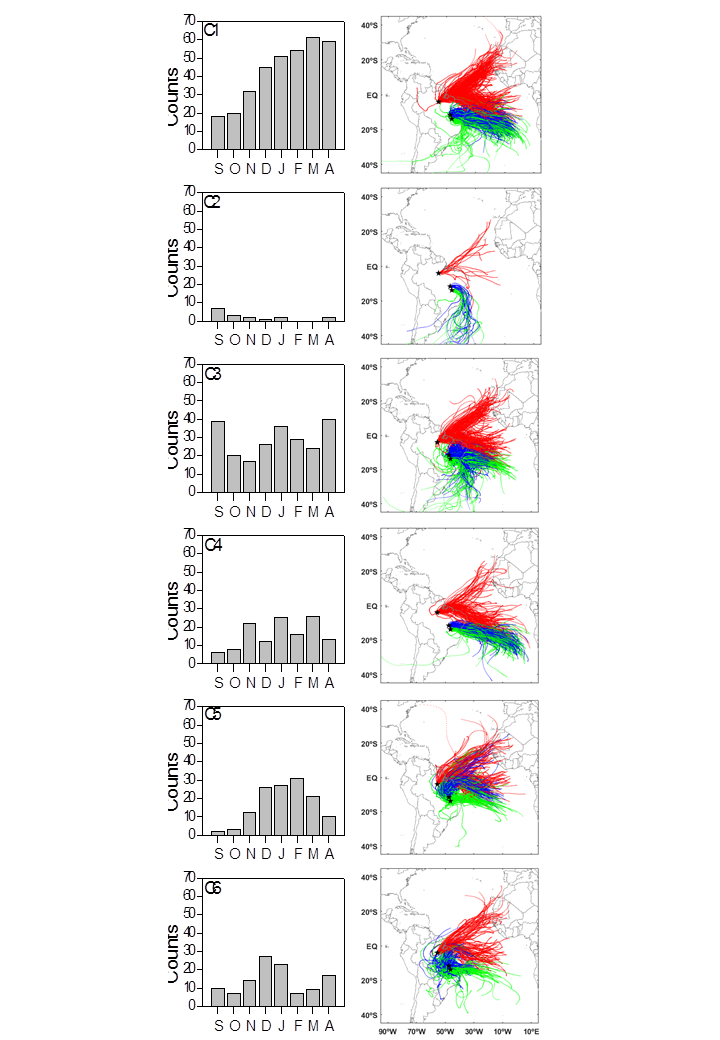


Figure S6. (Left) Bars represent the frequency of the main back-trajectory clusters per month. (Right) Paraíso (Red), Mata Virgem (Blue) and São Bernardo (Green) cave’s locations seven-day back trajectories from 2012 to 2018.

| **Sample** | | **^238^U** | | **^232^Th** | | | **^230^Th / ^232^Th** | | **δ^234^U*** | | | **^230^Th / ^238^U** | | **^230^Th Age (yr)** | | **^230^Th Age (yr)** | | **δ^234^U_Initial_**** | | **^230^Th Age (yr BP)***** | | **^230^Th Age (EC)** | | | |
| --- | --- | --- | --- | --- | --- | --- | --- | --- | --- | --- | --- | --- | --- | --- | --- | --- | --- | --- | --- | --- | --- | --- | --- | --- | --- |
| **Number** | | **(ppb)** | | **(ppt)** | | | **(atomic x10^-6^)** | | **(measured)** | | | **(activity)** | | **(uncorrected)** | | **(corrected)** | | **(corrected)** | | **(corrected )** | | **(corrected )** | | | |
| **MV3-10** | 1323 | | 2.3 | | 331 | 6.8 | 276 | 7.5 | 218 | 1.6 | 0.0042 | | 0.0001 | 375 | 6.7 | **370** | **8.0** | 218 | 1.6 | **303** | **±8** | | 1647 | ±8 |  |
| **MV3-48** | 3020 | | 4.8 | | 799 | 16.1 | 382 | 8.2 | 208 | 2.2 | 0.0061 | | 0.0000 | 554 | 4.3 | **548** | **6.2** | 208 | 2.2 | **479** | **±6** | | 1471 | ±6 |  |
| **MV3-95** | 2911 | | 7.7 | | 1085 | 22.0 | 337 | 7.2 | 218 | 1.8 | 0.0076 | | 0.0001 | 684 | 4.7 | **675** | **7.9** | 219 | 1.8 | **608** | **±8** | | 1342 | ±8 |  |
| **MV3-125** | 1661 | | 1.8 | | 281 | 5.9 | 1133 | 31.1 | 226 | 1.6 | 0.0116 | | 0.0002 | 1038 | 18.5 | **1034** | **18.7** | 226 | 1.6 | **965** | **±19** | | 985 | ±19 |  |
| **MV3-156** | 1932 | | 2.7 | | 1089 | 22.0 | 323 | 7.0 | 218 | 1.4 | 0.0110 | | 0.0001 | 992 | 8.2 | **979** | **12.6** | 219 | 1.4 | **912** | **±13** | | 1038 | ±13 |  |
| **MV3-205** | 1735 | | 2.4 | | 527 | 11.0 | 659 | 15.0 | 220 | 1.5 | 0.0121 | | 0.0001 | 1090 | 10.1 | **1082** | **11.3** | 221 | 1.5 | **1015** | **±11** | | 935 | ±11 |  |
| **MV3-215** | 1289 | | 1.3 | | 2243 | 45.0 | 223 | 4.8 | 227 | 1.4 | 0.0235 | | 0.0002 | 2110 | 16.5 | **2069** | **33.5** | 228 | 1.4 | **2000** | **±34** | | -50 | ±34 |  |

**Table S1.**U-Th dates from MV3 record. U decay constants: λ_238_ = 1.55125x10^-1010^ and λ_234_ = 2.82206x10^-611^. Th decay constant: λ_230_ = 9.1705x10^-611^. *δ^234^U = ([234U/238U]_activity_ – 1)x1000. ** δ^234^U_initial_ was calculated based on 230Th age (T), i.e., δ^234^U_initial_= δ^234^U_measured_ x e^λ234xT^. Corrected ^230^Th ages assume the initial ^230^Th/^232^Th atomic ratio of 4.4 ±2.2 x10^-6^. Those are the values for a material at secular equilibrium, with the bulk earth ^232^Th/^238^U value of 3.8. The errors are arbitrarily assumed to be 50%. ***B.P. stands for “Before Present” where the “Present” is defined as the year 1950 A.D.

| Lab. | Lab. Number | Sample | Depth  (cm) | Conventional radiocarbon age | Age calibrate |
| --- | --- | --- | --- | --- | --- |
| LACUFF | 170081 | XC01-2/20 | 20 | 456 ± 24 | 394 |
| BETA | 488006 | XC01-2/40 | 40 | 780±30 | 685 |
| BETA | 488007 | XC01-2/60 | 60 | 1700±30 | 1479 |
| BETA | 468959 | XC01-2/80 | 80 | 2920±30 | 2844 |

Table S2.^14^C dates, conventional ages and calibrated by the SHcal 13 curve.

Bibliography

1. Colinvaux, P. A., de Oliveira, P. E., Moreno, E. & Patiño, J. E. . *Amazon Pollen Manual and Atlas*. (Hardwood Academic Publishers, 1999).

2. Roubik, D. W. & Moreno, J. E. *Pollen and spores of Barro Colorado Island*. (Botanical Garden, 1991).

3. Lorente, F. L., Buso Junior, A. Á., de Oliveira, P. E. & Pessenda, L. C. R. *Atlas Palinológico*. (FEALQ, 2017).

4. Grim, E. C. & Troostheid, C. D. *Tilia 2.00, program for plotting palynological diagrams*. (Illinois State Museum, 1994).

5. Grim, E. C. *CONISS: Textbook of Pollen Analyses*. (Wiley & Sons, 1987).

6. Stein, A. F. *et al.* NOAA’s HYSPLIT Atmospheric Transport and Dispersion Modeling System. *Bull. Am. Meteorol. Soc.* **96**, 2059–2077 (2015).

7. Rolph, G., Stein, A. & Stunder, B. Real-time Environmental Applications and Display sYstem: READY. *Environ. Model. Softw.* **95**, 210–228 (2017).

8. Numaguti, A. Origin and recycling processes of precipitating water over the Eurasian continent: Experiments using an atmospheric general circulation model. *J. Geophys. Res. Atmos.* **104**, 1957–1972 (1999).

9. Blaauw, M. & Christeny, J. A. Flexible paleoclimate age-depth models using an autoregressive gamma process. *Bayesian Anal.* **6**, 457–474 (2011).

10. Jaffey, A. H., Flynn, K. F., Glendenin, L. E., Bentley, W. C. & Essling, A. M. Precision measurement of half-lives and specific activities of U235 and U238. *Phys. Rev. C* **4**, 1889–1906 (1971).

11. Cheng, H. *et al.* Improvements in 230 Th dating , 230 Th and 234 U half-life values , and U – Th isotopic measurements by multi-collector inductively coupled plasma mass spectrometry. *Earth Planet. Sci. Lett.* **371**–**372**, 82–91 (2013).
